# Acute kidney injury and acute kidney disease in high-dose cisplatin-treated head and neck cancer

**DOI:** 10.3389/fonc.2023.1173578

**Published:** 2023-06-09

**Authors:** Francesco Trevisani, Federico Di Marco, Giulia Quattrini, Nicola Lepori, Matteo Floris, Davide Valsecchi, Leone Giordano, Italo Dell’Oca, Sara Cardellini, Alessandra Cinque, Aurora Mirabile

**Affiliations:** ^1^ Department of Urology and Division of Experimental Oncology, Urological Research Institute (URI), IRCCS San Raffaele Scientific Institute, Milan, Italy; ^2^ Department of Medical Science and Public Health, University of Cagliari, Nephrology, San Michele Hospital, ARNAS G. Brotzu, Cagliari, Italy; ^3^ Emergency Department, IRCCS San Raffaele Scientific Institute, Milan, Italy; ^4^ Department of Otorhinolaryngology, IRCCS San Raffaele Scientific Institute, Milan, Italy; ^5^ Radiotherapy Department, IRCCS San Raffaele Scientific Institute, Milan, Italy; ^6^ Health Directorate, IRCCS Ospedale San Raffaele, Milan, Italy; ^7^ Biorek srl, San Raffaele Scientific Institute, Milan, Italy; ^8^ Department of Medical Oncology, IRCCS San Raffaele Scientific Institute, Milan, Italy

**Keywords:** AKI, acute kidney injury, cancer, cisplatin, head and neck, AKD, acute kidney disease

## Abstract

**Background:**

In locally advanced head and neck squamous cell carcinoma (LA-SCCHN) at least 200mg/m^2^ (standard dose 300 mg/m^2^) of cisplatin concomitant with radiotherapy represents the standard of care, both in postoperative and conservative settings. Nevertheless, high dose administration every 3 weeks is often replaced with low dose weekly cisplatin to avoid toxicities like kidney injury, though often failing to reach the therapeutic dose. Our aim was to investigate the incidence of renal impairment in the real-life setting, integrating high dose cisplatin with adequate supportive therapy, and to explore both Acute Kidney Injury (AKI) and Acute Kidney Disease (AKD), a recently described clinical renal syndrome that encompasses functional alterations of the kidney lasting fewer than 3 months.

**Methods:**

One hundred and nine consecutive patients affected by LA-SCCHN and treated with at least a cumulative dosage of 200 mg/m^2^ of cisplatin concomitant with radiotherapy were enrolled in this prospective observational study.

**Results:**

AKI was reported in 12.8% of patients, 50% of whom were stage 1 (KDIGO criteria), while 25.7% of the cohort developed AKD. Patients with baseline estimated Glomerular Filtration Rate (eGFR) < 90 ml/min showed a higher incidence of AKD (36.2% vs 17.7%). Hypertension, baseline eGFR, and therapy with Renin-angiotensin-aldosterone system inhibitors proved to be significant factors associated with both AKI and AKD.

**Conclusion:**

AKI and AKD are not rare complications of high-dose cisplatin, but an appropriate prevention strategy and accurate monitoring of patients during treatment could lead to a reduction of the burden of these conditions.

## 1 Introduction

Head and neck cancer represents the sixth most common neoplasm worldwide, accounting for 800,000 new cases and 400,000 deaths globally every year (1). Among head and neck cancers, squamous cell carcinoma is the most prominent and aggressive histology, being responsible for more than 90% of cases (2). The overall 5-year survival rate for people with locally advanced head and neck squamous cell carcinoma (LA-SCCHN) ranges from 33% to 68% depending on risk factors and primary site (3).

Due to the anatomically complex structure of the head and neck district, which is responsible for nourishment, language and breathing, a multidisciplinary clinical approach is always mandatory in order to keep the functional aspects intact. Therefore, the combination of surgery, chemotherapy and radiation are of paramount importance to maximize the efficacy of the therapy and minimize the rate of side effects (4).

Cisplatin is especially interesting since it has shown anticancer activity in a variety of tumors, including head and neck. In the 1960s it was found to have cytotoxic properties, and by the end of the 1970s it had earned a place as the key component in the systemic treatment of germ cell cancers. Among the many chemotherapy drugs that are widely used for cancer treatment, Cisplatin is one of the most compelling, and in 1978 it was the first FDA-approved platinum compound for treating cancer (5).

In day-to-day clinical practice, a schedule including at least two cycles of thrice-weekly high-dose administrations of cisplatin (100 mg/m^2^) given concomitantly with radiotherapy represents the standard of care for LA-SCCHN with a curative intent, both in postoperative and conservative settings.

However, due to the awareness of platinum-based, compound-related, renal toxicity, thrice-weekly high-dose cisplatin is often replaced with a lower-dose weekly schedule to avoid toxicities like kidney dysfunction (6).

Nevertheless, often this approach fails to reach the therapeutic cumulative dose of 200mg/m^2^, and since there is a paucity of prospective randomized studies, and the optimal dosing of weekly cisplatin is still a contentious issue, low dose weekly cisplatin should be prospectively studied in a phase III trial (7).

Moreover, several articles in the literature underline that the nephrotoxic effect of platin is cumulative and dose-dependent (8) (9). In fact, cisplatin accumulation in the kidneys increases the amount of tumor necrosis factor alpha and reactive oxygen species, resulting in inflammation, oxidative stress, vascular injury, and renal vasoconstriction (10). From a clinical point of view, all these biological processes promote the development of acute tubular necrosis and apoptotic cascade in the proximal tubules, inexorably leading to the onset of acute kidney injury (AKI). In addition, recurrent AKI events make a non-negligible percentage of patients susceptible to developing chronic renal impairment over time, thus resulting in chronic kidney disease (CKD) (11, 12).

However, despite all these nephrological considerations, from an oncological perspective, there is no level 1 evidence of comparable efficacy to cisplatin once every 3 weeks (13). Recently, a new clinical entity named Acute Kidney Disease (AKD), was proposed in order to consider all functional and structural kidney alterations lasting less than three months (14). The evaluation of AKD is of particular interest in LA-SCCHN patients to be able to explore the whole acute spectrum of renal damage after cumulative cisplatin exposure. Aim of this study was to investigate real-life acute nephrotoxicity in LA-SCCHN patients during and after treatment with high-dose cisplatin-based chemoradiotherapy (CRT), with particular focus on AKI and AKD onset.

## 2 Results

A total of 109 patients were included in this study. The mean age was 60 years (SD: 10) and 70.6% of patients were male. Mean estimated glomerular filtration rate (eGFR) at baseline was 90.8 ml/min/1.73m^2^: 57% of patients presented baseline eGFR ≥ 90 mL/min, while 43% showed < 90 mL/min (only 5 patients had eGFR < 60 mL/min). Median body mass index (BMI) was 24.8. Diabetic patients made up 9.2% of the total, while 36.7% had arterial hypertension. When patients with baseline eGFR ≥ 90 ml/min were compared to patients with eGFR < 90 ml/min, a statistically significant difference was found in both BMI (23.6 vs 25.3, p 0.003) and prevalence of arterial hypertension (22.6% vs 55.3%, p < 0.001) (**Table 1**).

**Table 1:** Descriptive statistics.

Parameter	eGFR ≥ 90 (N=62)	eGFR < 90 (N=47)	Total (N=109)	p value
Age (years)	55.8 (9.4)	64.6 (9.1)	59.6 (10.2)	< 0.001^1^
Gender				0.206^2^
F	15 (24.2%)	17 (36.2%)	32 (29.4%)	
M	47 (75.8%)	30 (63.8%)	77 (70.6%)	
Cisplatin cumulative dose (mg/m^2^); Mean (SD)	254.9 (57.7)	248.4 (65.6)	252.1 (61.1)	0.585^1^
sCr (mg/dl); Mean (SD)	0.7 (0.1)	1.0 (0.2)	0.8 (0.2)	< 0.001^1^
eGFR (ml/min/1.73^2)^; Median (SD)	102.4 (10.0)	75.6 (12.0)	90.8 (17.2)	< 0.001^1^
BMI;Mean (SD)	23.5 (3.9)	25.9 (4.3)	24.5 (4.2)	0.003^1^
Diabetes	4 (6.5%)	6 (12.8%)	10 (9.2%)	0.323^2^
Hypertension	14 (22.6%)	26 (55.3%)	40 (36.7%)	<0.001^2^
ASA	6 (9.7%)	2 (4.3%)	8 (7.3%)	0.462^2^
PPI	7 (11.3%)	8 (17.0%)	15 (13.8%)	0.414^2^
Steroids	0 (0.0%)	1 (2.1%)	1 (0.9%)	0.431^2^
AKI (increase in sCr > 50%)	11(17.8%)	3 (6.4%)	14 (12.8%)	0.0912^2^
AKD (AKI or eGFR < 60 ml/min/1.73 m^2^, or eGFR decrease by ≥ 35% or s-Cr increase by 50% occurring from less than 90 days)	11 (17.7%)	17 (36.2%)	28 (25.7%)	0.045^2^

Legend 1: T-test; Legend 2: Fisher’s exact test; SD, standard deviation; sCr, serum creatinine; eGFR, estimated Glomerular Filtration Rate; BMI, body mass index; ASA, acetylsalicylic acid treatment; PPI, proton pump inhibitors; RAAS, Renin-angiotensin-aldosterone system; AKI, acute kidney injury; AKD, acute kidney disease.

Forty patients (38.8%) were on anti-hypertensive medication: no differences were found between patients with baseline eGFR ≥ 90 ml/min and those with eGFR < 90 ml/min (**Table 2**).

**Table 2:** Anti-hypertensive drug use by the study cohort.

Parameter	≥ 90 (n=14)	< 90 (n=26)	Total (n=40)	p value
RAAS inhibitors				0.501^1^
0	6 (42.9%)	8 (30.8%)	14 (35.0%)	
1	8 (57.1%)	18 (69.2%)	26 (65.0%)	
Beta blockers				1.000^1^
0	10 (71.4%)	19 (73.1%)	29 (72.5%)	
1	4 (28.6%)	7 (26.9%)	11 (27.5%)	
Calcium antagonists				0.350^1^
0	13 (92.9%)	26 (100.0%)	39 (97.5%)	
1	1 (7.1%)	0 (0.0%)	1 (2.5%)	
Diuretics				0.453^1^
0	12 (85.7%)	19 (73.1%)	31 (77.5%)	
1	2 (14.3%)	7 (26.9%)	9 (22.5%)	

Legend: 1. Fisher’s exact test; RAAS, Renin-angiotensin-aldosterone system.

Acute Kidney Injury was reported in 14 patients (12.8%): the majority of AKI episodes (50%) were stage 1 according to KDIGO criteria, 43% were AKI stage 2 and only 1 patient had AKI stage 3. Patients with baseline eGFR ≥ 90 ml/min/1.73m^2^ showed a higher incidence of AKI (17.8% vs 6.4%) when AKI was defined as an increase in serum creatinine (sCr) >50% of baseline, although the difference was not statistically significant (p=0.091). Median cumulative cisplatin dose was 300 mg/m^2^ (interquartile interval 200-300): 99 patients (90.8%) reached a cisplatin-cumulative dose >200 mg/m^2^.

Acute Kidney Disease occurred in 28 patients (25.7%). Patients with baseline eGFR ≥ 90 ml/min/1.73m^2^ showed a statistically significant lower incidence of AKD (17.7% vs 36.2%; p=0.045).

Median cumulative cisplatin dose was 300 mg/m^2^ (interquartile interval 200-300): 99 patients (90.8%) reached a cisplatin-cumulative dose >200 mg/m^2^. Five patients discontinued planned cisplatin-based chemotherapy due to sepsis (1 patient), AKI (1 patient), cytopenia (2 patients), hypoacusis (1 patient) and death due to other causes (1 patient).

In order to better define which aspect might be most involved in eGFR decline as a predictive risk factor during therapy, we decided to use a multivariable linear regression model for eGFR percentage decay, including comorbidities, baseline eGFR, cycle treatment and number of days from last cycle. The analysis showed the presence of hypertension (beta = 3%, CI=0.2%-6%, p=0.03), baseline eGFR (beta = 0.1%, CI = 0.05%-0.2%, p=0.001) and the number of chemotherapy cycles (beta = 3%, CI = 2%-4%, p < 0.001) to be predictive factors (**Supplementary Data Table 1**).

In order to evaluate which clinical and demographic factors were related to the risk of developing AKI during cisplatin treatment, a multivariable logistic analysis was performed. Results showed a significant correlation for baseline eGFR (OR = 1.03, CI=1;1.1, p=0.04), hypertension (OR=3.26, CI=1.4;7.6, p=0.005), number of chemotherapy cycles (OR=1.82, CI=1.2;2.9, p=0.007) and number of days after the last cycle (OR=1.15, CI=1.1;1.3, p < 0.001). The same analysis was performed in order to evaluate the factors linked to AKD development. Despite AKI results, an inverse correlation between baseline eGFR and AKD was found (OR = 0.95, CI=0.92;0.97, p=0.04). A significant correlation was also found with hypertension (OR=4.95, CI= 1.8;14, p=0.002), and the number of days after the last cycle (OR=1.09, CI=1;1.2, 0.02) (**Table 3**).

**Table 3:** Multivariable logistic regression model: AKI onset.

Parameter	AKI			AKD		
	Odds Ratio	CI	p	Odds Ratio	CI	p
Baseline eGFR	1.03	1;1.1	0.04	0.95	0.92;0.97	<0.001
Hypertension	3.26	1.4;7.6	0.005	4.95	1.8;14	0.002
Diabetes	1.38	0.37;4.2	0.6	1.76	0.49;5.8	0.4
RAAS inhibitors	0.13	0.033;0.44	0.002	0.29	0.1;0.83	0.02
BMI	0.96	0.88;1.1	0.4	0.93	0.84;1	0.09
Age	1.02	0.98;1.1	0.4	1.03	0.98;1.1	0.2
Gender (Male)	1.14	0.51;2.8	0.8	0.79	0.36;1.7	0.5
Chemo Cycle	1.82	1.2;2.9	0.007	1.63	0.81;3.3	0.2

RAAS, Renin-angiotensin-aldosterone system.

## 3 Discussion

Cisplatin-based chemotherapy is widely used in the management of locally advanced squamous cell carcinoma of the head and neck to enhance the tumoricidal activity of irradiation, adding to radiotherapy alone a survival benefit estimated at 6.5% at 5 years. AKI is a common adverse effect associated with cisplatin CRT and represents a possible limitation to the delivery of the planned cisplatin dose. Moreover, AKI is a well-known risk factor for the development of CKD.

The present cohort study shows that 12.8% of 109 patients with head and neck cancer treated with high-dose cisplatin developed AKI according to the KDIGO definition and staging criteria. The majority of AKI episodes (50%) were stage 1, 43% were AKI stage 2 and only 1 patient had AKI stage 3. Interestingly, only 1 patient among those who experienced AKI (AKI stage 3) discontinued cisplatin-based treatment. Among the several potential risk factors studied (use of potentially nephrotoxic drugs, use of angiotensin converting enzyme inhibitors [ACEi], hypertension, diabetes, number of CT cycles, baseline eGFR), predictive risk factors for cisplatin-induced AKI included hypertension, and number of chemotherapy cycles. Previous studies in patients affected by LA-SCCHN showed broad variability in AKI incidence during cisplatin-based CRT, ranging from 4% to 69% (**Table 4**).

**Table 4:** Main studies on AKI and AKD incidence in head and neck cancer patients treated with cisplatin-based chemotherapy.

Authors	Year	Patients	Cisplatin schedule	AKI-AKD definition	AKI-AKD incidence	CT stop due to AKI (%)	AKI Risk-Factors
(6)	2019	124	100 mg/m^2^ thrice-weekly	KDIGO criteria	69%	22.6%	Hypertension and CINV
(15)	2015	287	40 mg/m^2^ weekly	CTCAE 3.0	18.8%	Not known	Not known
(16)	2020	246 (> 65y/o)	40 mg/m^2^ weekly **OR** 100 mg/m^2^ thrice-weekly	CTCAE 5.0	4.06%	Not Known	Low BMI
(17)	2015	144	40 mg/m^2^ weekly (104 pts) **VS** 100 mg/m^2^ thrice-weekly (40 pts)	RIFLE criteria	7%	0% (40 mg/m^2^/week) **VS** 18% (100 mg/m^2^ thrice-weekly)	High cisplatin dose
(18)	2018	82	100 mg/m^2^ thrice-weekly	KDIGO criteria	34%	6%	Low serum creatinine, low BMI
(19)	2015	233	100 mg/m^2^ thrice-weekly	KDIGO criteria	68%	Not known	African- American ethnicity
(20)	2006	62	100 mg/m^2^ thrice-weekly	Increase in sCr > 0.5 mg/dl	40%	Not known	Treatment with multivitamin, high serum calcium levels, African-American ethnicity
(21)	2015	173	Not specified	AKIN criteria	52.9%	Not known	Weight loss > 10%, ACEi treatment
(22)	2020	509	75–100 mg/m^2^ every 3 weeks for up to 3 cycles OR 40mg/m^2^ weekly for up to 7 cycles	KDIGO criteria	13.4%	0%	Unknown AKI risk factors. Hypertension was a risk factor for AKD
(23)	2022	30	40 mg/m2 weekly for 7-8 weeks	KDIGO criteria	3%	0%	Unknown
(24)	2021	120	30-40 mg/m^2^ weekly	KDIGO criteria (AKD)	38.4% (AKD)	Not known	Unknown

CINV, chemotherapy-induced nausea and vomiting; CTCAE, Common Terminology Criteria for Adverse Events; AKD, Acute Kidney Disease (renal impairment within 3 months after receiving the renal insult).

The explanations for this broad difference in AKI incidence reside mainly in the use of different definitions of AKI in different studies (**Table 5**). Studies which used different versions of the Common Terminology Criteria for Adverse Events system (CTCAE), i.e., CTCAE 3.0, CTCAE v5.0, showed the lowest incidence of AKI (15, 16). The 3.0 version of the CTCAE system defines AKI as an increase in sCr to a level higher than the upper limit of normal for the specific laboratory, while with regard to the CTCAE 4.0 version, an increase in sCr >0.3 mg/dl has been introduced as an AKI diagnostic criterion, following the KDIGO definition of AKI (6). However, in contrast to KDIGO criteria, there is no provision of a time course in CTCAE versions 4.0 and 5.0. Consequently, the higher AKI incidence observed in those studies which used KDIGO criteria or AKIN criteria (6) is likely due to a more frequent diagnosis of early stages of AKI. Interestingly, a correlation between cisplatin doses used in cisplatin-based CRT and incidence of AKI has been reported in several studies. Driessen and colleagues showed that 7% of patients who underwent intermediate-dose cisplatin-based CRT (40 mg/m^2^ weekly) developed AKI (all stage 1 according RIFLE criteria), while a much higher incidence of AKI (70%) was described in the high-dose group (100 mg/m^2^ thrice-weekly) (17). These results are coherent with a retrospective study published by Rades in 2011 which found a significant difference in grade 3 nephrotoxicity (using the CTCAE 2.0 version) between patients treated with cisplatin 100 mg/m^2^ on days 1, 22, and 43 and cisplatin 20 mg/m^2^ on days 1 to 5 and 29 to 33 of 8% versus 1% (29).

**Table 5:** Functional criteria utilized for acute kidney injury and acute kidney disease definition in head and neck cancer patients.

RIFLE-AKI (25) Rise in sCr within 7 days	AKIN-AKI (26) Rise in sCr within 48 h	CTCAE-AKI (version 5.0) (27) A finding based on laboratory test results that indicate increased sCr levels	KDIGO-AKI (28)* sCr increase≥ 0.3 mg/dL within 48 h or Increase in sCr ≥ 1.5 × baseline that is known or presumed to have occurred within the past 7 days	KDIGO-AKD (24)* Rise in sCr within 3 months
**Risk:** Increased sCr x 1.5 or GFR decrease > 25%	**Stage 1**: Same as RIFLE-Risk or sCr increase ≥ 0.3 mg/dL (≥ 26.4 μmol/L)	**Grade 1**: sCr >ULN - 1.5 x ULN	**Stage 1**: 1.5-1.9 × baseline sCr or ≥ 0.3-mg/dL increase in baseline sCr	AKI or eGFR decrease < 60 ml/min/1.73 m^2^, or eGFR decrease by ≥ 35% or eGFR increase by 50% occurring from less than 90 days
**Injury:** Increased sCr x 2 or GFR decrease > 50%	**Stage 2**: Same as RIFLE-Injury	**Grade 2**: >1.5 - 3.0 x baseline; >1.5 - 3.0 x ULN	**Stage 2**: 2.0-2.9 × baseline sCr	
**Failure:** Increased sCr x 3 or GFR decrease 75% or sCr ≥ 4 mg/dL	**Stage 3**: Same as RIFLE-Failure or RRT initiation	**Grade 3**: >3.0 x baseline; >3.0 - 6.0 x ULN	**Stage 3**: 3.0 × baseline sCr or sCr increase to ≥ 4.0 or RRT initiation	
**Loss:** complete loss of kidney function > 4 weeks		**Grade 4**: >6.0 x ULN		
**End Stage:** ESRD (> 3 months)				

*criteria utilized in our research.

RIFLE, Risk of renal failure, injury to the kidney; AKI, Acute Kidney Injury; sCr, Serum creatinine; GFR, Glomerular filtration rate; ESRD, End-stage Renal Disease; AKIN, Acute Kidney Injury Network; RRT, Renal replacement therapy; CTCAE, Common Terminology Criteria for Adverse Events; ULN, upper limit normal, failure of kidney function, loss of kidney function, and end-stage renal failure; eGFR, estimated Glomerular Filtration Rate.

Although low-dose, once-a-week cisplatin could be considered a promising strategy to attempt to avoid AKI, often this approach fails to reach the therapeutic cumulative dose of 200 mg/m^2^ and, since there is a lack of prospective randomized studies, the optimal dosing of weekly cisplatin is still a contentious issue (7). Reported predictors of cisplatin-induced AKI included older age and hypertension, female sex, smoking, black ethnicity, hypokalemia, and hypoalbuminemia (30). Data from LA-SCCHN patients showed several risk factors for cisplatin-induced AKI: hypertension (6), low BMI (16, 18), low sCr (18), African-American ethnicity (19), treatment with ACEi (21). In our study, consistently with data present in the literature, hypertension was the most important factor associated with increased risk of cisplatin-induced AKI. Hypertension is a well-known risk factor for AKI in cisplatin-based chemotherapy, as described by several authors (31, 32). The most likely explanation for the correlation between hypertension and increased risk of cisplatin-nephrotoxicity is the presence of subclinical kidney damage due to hypertension, which could constitute a predisposing ground for cisplatin nephrotoxicity (31). In fact, hypertension can cause ischemic glomerulosclerosis, which further stimulates interstitial fibrosis and atrophy of tubular cells, the main target of cisplatin nephrotoxicity (33).

Acute Kidney Disease is a term that was introduced in 2017 to define all renal function alterations not fulfilling AKI and CKD criteria: the current definition includes AKI or eGFR < 60 ml/min/1.73 m^2^, or eGFR decrease by ≥ 35% or sCr increase by 50% occurring from less than 90 days (14). Few studies have evaluated the incidence of AKD in the setting of high-dose cisplatin chemotherapy in LA-SCCHN. In 2021, Patimarattananan et al. published a retrospective study analyzing data from 509 patients. Reported incidence of AKI and AKD were respectively 13.4 and 27.9%. On multivariate analysis, hypertension was found to be related to long term follow-up of these patients: patients who were diagnosed with AKD showed a more significant decline in eGFR at 3 months (-39.4% from baseline) without complete recovery of pre-CRT renal function at 12 months (-29.1% from baseline) (22). A higher incidence of AKD (38%) was demonstrated by an Indonesian group that analyzed data from a cohort of 120 patients with local advanced nasopharyngeal cancer treated with cisplatin-based CRT (24). Our data showed an incidence of AKD of 25.7% (similar to Patimarattanan et al, but lower than data provided by Rachman and colleagues), clearly demonstrating 2 risk factors for AKD: hypertension and baseline eGFR < 90 ml/min.

In this scenario, our study shows a lower incidence of AKI compared to previously published studies which used KDIGO or RIFLE criteria to define AKI, in which AKI incidence ranged from 34% to 69% (6, 19). The reasons for this finding are not completely clear. Although a role of the different composition of the studied populations cannot be excluded, it seems unlikely, since baseline features of our cohort do not differ significantly from those of cohorts described in other studies (**Table 6**). Moreover, mean age, prevalence of female sex and prevalence of diabetes are higher in our cohort than in the populations described in other studies.

**Table 6:** Comparison among studies which used KDIGO criteria to define AKI or AKD in the setting of head and neck cancer patients treated with cisplatin-based CRT.

Study	Year	N.	Cisplatin- schedule	Age	F sex (%)	Htn (%)	DM (%)	Baseline SCr (mg/dl)	Baseline eGFR (ml/min)
Our study	2022	109	100 mg/m^2^ thrice-weekly	60	29.4	36.7	9	0.8	90.8
(18)	2018	82	100 mg/m^2^ thrice-weekly	54	23	44	7	0.86	94.9
(6)	2019	124	100 mg/m^2^ thrice-weekly	60	22	20	7	0.75	Not Known
(19)	2015	233	100 mg/m^2^ thrice-weekly	53.6	24	41	7	Not known	117
(22)	2020	509	75–100 mg/m^2^ every 3 weeks for up to 3 cycles **OR** 40mg/m^2^ weekly for up to 7 cycles	56*	28.9*	14.8*	21*	0.79*	97*
(24)	2021	120	30-40 mg/m^2^ weekly	45.1*	25.8*	25.8*	Not known	Not known	102*
(23)	2022	30	40 mg/m2 weekly for 7-8 weeks	52.2	23.3	13.3	20	0.78	99

F sex, female sex; Htn, hypertension; DM, diabetes mellitus; *AKD patients, Acute Kidney Disease (renal impairment within 3 months after receiving the renal insult).

Therefore, a possible cause of the low incidence of AKI in our cohort could be the rigorous protocol used in our study to prevent cisplatin-induced nephrotoxicity. All patients received intravenous hydration with 0.9% saline 750 ml + 16mEq of magnesium sulfate before and after cisplatin administration (a total of 1500 ml at day 0 and again at day 1). Mannitol 10% (375 ml) was administered if diuresis after hydration was less than 100 ml/hour; meanwhile antiemetic prophylaxis (to prevent chemotherapy-induced nausea and vomiting which could lead to hypovolemia and an increased risk of AKI) with dexamethasone 12 mg IV as chemo premedication at day 0 and 8 mg PO at days 1 to 3 was administered. Evidence from previous studies is not strong enough to support the superiority of a specific hydration regimen for preventing nephrotoxicity associated with cisplatin administration.

Hypomagnesemia can upregulate OCT-2, leading to increased cisplatin transport to the kidneys, thus resulting in nephrotoxicity (34). Several systematic reviews suggest that magnesium supplementation may limit cisplatin-induced nephrotoxicity (35). A recent study by Kimura et al, analyzed the nephroprotective role of magnesium supplementation in patients receiving cisplatin for head and neck cancer: in multivariate analysis the use of magnesium-supplemented hydration was the only factor associated with a reduction in AKI-incidence (OR 0.157) (36). The use of mannitol to promote diuresis (and consequent cisplatin clearance) could raise concerns regarding over-diuresis and possible pre-renal AKI due to dehydration. Data from different studies are conflicting. A retrospective study conducted among head and neck tumor patients highlighted that patients who did not receive mannitol were more likely to develop nephrotoxicity (37), while Leu and colleagues showed a higher incidence of AKI in patients who did receive mannitol (38). In order to prevent potential over-diuresis, in our protocol mannitol was used only in patients who did not reach adequate diuresis after hydration.

A result of non-univocal interpretation regards the role of ACEi/angiotensin receptor blocker (ARB) treatment. Previous studies showed an increased risk of AKI in patients receiving ACEi therapy. Our study does not show this correlation: the risk of AKD appears lower in patients treated with ACEi/ARBs. The meaning of this result is not clear: a possible explanation could be that the hydration protocol and the active management of ACEi/ARBs (discontinuation of ACEi/ARBs in the most frail patients) may have reduced the incidence of AKD in this subset of patients. The lack of correlation between the increased risk of AKD and the use of ACEi/ARBs represents a relevant clinical finding, supporting the hypothesis that in our cohort, the main determinants of the development of AKD were renal function at the time of chemotherapy initiation, hypertension and the chemotherapy treatment itself.

An important result of our study is the extremely low incidence of stage 3 AKI and AKD (1 case). Similar data have been previously described by several authors. Van der Vorst reported stage 3 only in 5% of AKI cases, while in the study published by Faig in 2015, among 88 patients only 4 cases of stage 3 AKI were reported (6, 18). The low incidence of severe AKI in our cohort is confirmed by the evidence that only 1 patient out of 109 discontinued cisplatin-based chemotherapy because of nephrotoxicity.

The concept of AKD is relatively new in nephrology literature, and data on the incidence of AKD in cancer patients are still scarce (22, 24, 34). The evidence that this pathological entity is significantly correlated with renal prognosis in the mid to long term (progression to chronic kidney disease, end-stage renal disease and death), makes the possibility of using it to evaluate the impact of chemotherapy treatment on renal function very attractive. The higher incidence of AKD compared to AKI in our cohort (data also confirmed by previously published studies) could be an expression of repeated exposure to chemotherapy treatment leading to a progressive worsening of renal damage (not always of such a degree as to allow us to identify a picture of AKI). In our opinion, the use of the AKD concept is very promising as it allows the clinician to intercept those conditions of renal damage that do not fall within the AKI criteria. The prevalence of AKD we have demonstrated makes it clear that a non-negligible percentage of patients undergoing chemotherapy with cisplatin develop renal impairment that would not be recognized using the classic criteria for AKI. These patients represent a population of cancer patients worthy of long-term nephrological follow-up considering the risk of progression to CKD and end-stage renal disease.

A second significant clinical message from these results is that although cisplatin-induced AKI and AKD are not rare events (12.8% and 25.7% of patients in our cohort), severe AKI and AKD are rare, and in the vast majority of cases the development of AKI or AKD does not affect the achievement of the planned cisplatin dose.

Our study presents some limitations but also several strengths. The main strengths of this study are the size and the careful characterization of the study cohort, as well as the homogeneity of treatment, prophylactic measures and clinical management of patients. Another strength of our study is the identification of several risk factors for cisplatin-induced AKI and AKD. The main limitation is the absence of long-term follow-up, which does not allow to evaluate the clinical meaning of the AKI and AKD episode as it relates to renal function and patient survival. Moreover, data regarding urinary output (which is part of the AKI definition according to KDIGO criteria), markers of tubular injury (urinary b-2 microglobulin, urinary bicarbonates, urinary ph), and proteinuria levels are not available. Other weaknesses include; the relatively small percentage of patients receiving ACEi and ARB treatment, which could lead to underestimation of the role of these medications in increasing the risk of renal damage, the single-center nature of the study, the absence of evaluation of quality of life in our patients.

## 4 Materials and methods

All 109 consecutive adult patients treated with cisplatin-based CRT for LA-SCCHN between January 2017 and December 2021 were enrolled in a prospective, single-center study in a tertiary care university hospital. Inclusion criteria were: age above 18 years, complete availability of clinical and laboratory data at the time of CRT start, informed consent. Exclusion criteria were: absence of complete clinical and laboratory data, age below 18 years, refusal to sign informed consent, a diagnosis of end-stage renal disease. The follow-up period was in line with the duration of the treatment (3 cycles), and continued until discontinuation due to adverse events requiring treatment schedule modification and/or discontinuation, or death.

All patients received adequate supportive care including 750 ml of saline solution + 16mEq of intravenous magnesium sulphate infused at the rate of 200 ml/h before and after cisplatin administration (a total of 1500 ml at day 0 and again at day 1). In patients with diuresis less than 100 ml/hour after hydration, 375 ml of 10% mannitol was administered. Lastly, dexamethasone at a dose of 12 mg was administered intravenously to all patients as chemo premedication on day 0, and orally at a dose of 8 mg on days 1 to 3 as antiemetic prophylaxis, followed by six days of progressive tapering. The present study was performed in accordance with the Declaration of Helsinki (6^th^ revision, 2008), and the study protocol was reviewed and approved by the local Ethics committee.

### 4.1 Data collection

Demographic data, medical history, and clinical, laboratory, and histological data at presentation were retrieved from the medical records. For this study we considered the following data: age, gender, BMI, TNM staging, comorbidities (hypertension, diabetes, cardiovascular disease, thyroid disease), platinum dose, and medical therapy (ACEi, ARBs), calcium antagonists, beta blockers and diuretics). Use of concomitant medications that have been reported to cause acute interstitial nephritis, such as non-steroidal anti-inflammatory drugs, allopurinol, proton pump inhibitors and antibiotics, as well as contrast agents for CT-scan or MRI, were reported before treatment and during follow up. Patients were evaluated at the time of the clinical visit in order to identify known causes of renal damage other than cisplatin exposure; these patients were excluded from the analysis. Serum creatinine values (Kinetic Picrate standardized (COBAS C 800) for IDMS) were recorded at baseline and after each cycle of treatment, respectively, at day 1 and day 7. The eGFR was calculated using the creatinine-based eGFR formula, CKD-EPI 2012.

Chronic Kidney Disease classes were created according to the KDIGO guidelines for G categories with the following thresholds: over 90 mL/min/1.73m^2^ (G1), 89-60 mL/min/1.73m^2^ (G2), 59-45 mL/min/1.73m^2^ (G3a), 44-30 mL/min/1.73m^2^ (G3b) and below 30 mL/min/1.73m^2^ (G4-5) (39). Acute kidney injury onset was diagnosed according to KDIGO 2012 criteria, based on the sCr increase, i.e., a sCr increase greater than 1.5 times the baseline value was classified as stage 1, greater than 2 times as stage 2, and greater than 3 times as stage 3 (28). Urinary output data were not collected for AKI evaluation. In 2017, the Acute Disease Quality Initiative (ADQI) and KDIGO consensus conferences introduced the term ‘Acute Kidney Disease’ in an effort to define all renal function alterations not fulfilling AKI and CKD criteria. Although the original definition encompassed all renal function abnormalities occurring from 7 to 90 days following a possible insult (40), the current definition includes AKI or eGFR < 60 ml/min/1.73 m^2^, or eGFR decrease by ≥ 35%, or sCr increase by 50% occurring less than 90 days after the possible insult (24). In our manuscript, all episodes of a sCr increase of more than 50% within 7 days after cisplatin exposure have been defined as AKI, while the AKD term was reserved for renal function abnormalities occurring up to 90 days after cisplatin exposure.

### 4.2 Statistical analysis

Comparisons between numerical variables were performed using multivariable linear regressions to evaluate clinical factors associated with eGFR decline; for between-group differences, the t-test for numerical variables was used, while Fisher’s exact test was used for categorical variables. Multivariable logistic regression was used to evaluate clinical factors related to AKI onset. Data analysis was performed using programming language R and RSTUDIO integrated development environment.

## 5 Conclusions

In literature, AKI and AKD are frequent and frightening complications of high-dose cisplatin-based CRT in patients with LA-SCCHN, thus limiting the use of this type of chemotherapy in the general population and especially in CKD patients.

Our study highlights a lower incidence of platinum-induced AKI than previously reported, with the presence of hypertension and normal baseline eGFR ≥ 90 ml/min/1.73 m^2^ as possible risk factors for both AKI and AKD development. Moreover, hydration and magnesium support represent the cornerstone of AKI prevention in our cisplatin-based CRT, suggesting that both patients with normal and mildly reduced renal function could undergo high dosage cisplatin therapy for LA-SCCHN as long as monitoring with an accurate multidisciplinary strategy is carried out. Further studies are needed to confirm our results.

## Data availability statement

The raw data supporting the conclusions of this article will be made available by the authors, without undue reservation.

## Ethics statement

The studies involving human participants were reviewed and approved by San Raffaele Ethics Committee (65/INT/2022). Informed consent was obtained from all subjects involved in the study.

## Author contributions

Conceptualization: FT, AM, MF, and NL; Methodology: FT, FM, MF, NL, and AM; Investigation: GQ, DV, LG, ID’O, and SC; Visualization: FT, and MF; Supervision: FT, MF, AM, and NL; Project administration: FT, AC, and AM; Software: FM; Formal analysis: FM; Data curation: FM, NL, and GQ; Writing – original draft: FT, AM, NL, and MF; Writing – review and editing: FT, NL, MF, and AM. All authors contributed to the article and approved the submitted version.
