# Supplementary material for: Acute kidney injury and acute kidney disease in high-dose cisplatin-treated head and neck cancer
Source: Front Oncol. 2023 Jun 9;13:1173578. doi: 10.3389/fonc.2023.1173578 (PMC10289148; doi:10.3389/fonc.2023.1173578)
Supplement: Supplementary file 1 [file Table_1.docx]

| ID | b | CI | p |
| --- | --- | --- | --- |
| Basal eGFR | 0.1 | 0.05;0.2 | 0.001 |
| Hypertension | 0.5 | -3;4 | 0.8 |
| RAAS inhibitors1 | 4.0 | -0.4;8 | 0.08 |
| Diabetes | 2.0 | -3;7 | 0.4 |
| BMI | -0.1 | -0.4;0.1 | 0.3 |
| Chemo Cycle | 3.0 | 2;4 | <0.001 |

Supplementary data - table 1 – Multivariable linear regression model: eGFR percentage decay
